# Cardiomyocyte Specific Deletion of Crif1 Causes Mitochondrial Cardiomyopathy in Mice

**DOI:** 10.1371/journal.pone.0053577

**Published:** 2013-01-04

**Authors:** Juhee Shin, Seok Hong Lee, Min-Chul Kwon, Dong Kwon Yang, Ha-Rim Seo, Jaetaek Kim, Yoon-Young Kim, Sun-Kyoung Im, Evan Dale Abel, Kyong-Tai Kim, Woo Jin Park, Young-Yun Kong

**Affiliations:** 1 Department of Biological Sciences, Seoul National University, Gwanak-gu, Seoul, Republic of Korea; 2 Department of Life Sciences, Pohang University of Science and Technology, Pohang, Kyungbuk, Republic of Korea; 3 Division of Endocrinology and Metabolism, Department of Internal Medicine, College of Medicine, Chung-Ang University, Dongjak-gu, Seoul, Republic of Korea; 4 Global Research Laboratory and Department of Life Science, Gwangju Institute of Science and Technology, Gwangju, Republic of Korea; 5 Program in Molecular Medicine, University of Utah School of Medicine, Salt Lake City, Utah, United States of America; Université Joseph Fourier, France

## Abstract

Mitochondria are key organelles dedicated to energy production. Crif1, which interacts with the large subunit of the mitochondrial ribosome, is indispensable for the mitochondrial translation and membrane insertion of respiratory subunits. To explore the physiological function of Crif1 in the heart, *Crif1^f/f^* mice were crossed with *Myh6-cre/Esr1* transgenic mice, which harbor cardiomyocyte-specific Cre activity in a tamoxifen-dependent manner. The tamoxifen injections were given at six weeks postnatal, and the mutant mice survived only five months due to hypertrophic heart failure. In the mutant cardiac muscles, mitochondrial mass dramatically increased, while the inner structure was altered with lack of cristae. Mutant cardiac muscles showed decreased rates of oxygen consumption and ATP production, suggesting that Crif1 plays a critical role in the maintenance of both mitochondrial structure and respiration in cardiac muscles.

## Introduction

Mitochondria are dynamic organelles performing various cellular functions, such as energy production, fatty acid/amino acid oxidation, iron metabolism and apoptosis [1]. According to a recently defined mitochondrial protein inventory, MitoCarta, there are 1098 mitochondrial proteins in mouse [2]. Only thirteen subunits of the respiratory chain complexes are encoded in the mitochondrial genome (MtDNA), and the rest of the mitochondrial proteins are encoded in the nuclear genome (NuDNA) [3], [4]. MtDNA encoded proteins are synthesized in the mitochondria by using its own transcriptional and translational machineries, whereas the NuDNA encoded precursor proteins are synthesized in the cytosol, imported into mitochondria, and processed into mature proteins [1], [4]. About 600 mitochondrial proteins remain without known function or only with domain predictions based on sequence homology [2], [3]. Thus, further investigations are necessary to identify the function of individual proteins constituting the mitochondrial proteome.

Crif1 had been recognized as a nuclear protein acting as a coactivator of transcriptional factors such as STAT3 and Elf3 [5], [6], [7], [8], [9], until it was identified as one of the MitoCarta genes [2]. Former studies used N-term tagged Crif1 for experiments, and the exogenous Crif1 was observed exclusively in the nucleus [5], [6], [7], [8], [9]. However, it was recently discovered that Crif1 has a signal peptide at the N terminus, and endogenous Crif1 is mostly located in the mitochondria. As a novel interacting factor of the mitoribosomal large subunit, Crif1 is indispensible for mitochondrial translation and membrane insertion of mtDNA encoded respiratory chain subunits. The targeted loss of Crif1 in mouse fibroblasts impaired energy generation and caused cell death. In addition, Crif1 loss in mouse forebrain induces neurodegeneration associated with mitochondrial abnormalities [10]. Crif1 is detected in the mitochondria of diverse mouse organs including cardiac and skeletal muscle, brain, kidney, liver, stomach and intestines [2]. Taken together, Crif1 appears to be a potential target for tissue-specific gene ablation to generate animal models of mitochondrial dysfunction.

Mitochondrial alterations have been implicated in a wide variety of disorders. Especially, defective mitochondrial respiration or oxidative phosphorylation causes mitochondrial respiratory disorders, which have an estimated occurrence of 1∶5000 live births, but yet with no curable treatments [11], [12], [13], [14]. Genetic mutations in the mitochondrial as well as the nuclear genome cause the mitochondrial respiratory disorder, and more than 100 causal genes have been reported [15]. The range of clinical manifestations is extensively broad concerning the affected organs, onset age, symptoms, and severity. Multiple defects in different organs are common, and most vulnerable tissues include the nervous, muscle and cardiac tissues, of which cell types require high energy metabolism [12], [14]. Mitochondrial cardiomyopathy (MCM), a common manifestation of mitochondrial respiratory disorders, involves the development of cardiac hypertrophy and heart failure [16], [17]. The age of onset for MCM is variable, as it can be detected in infants, children or adults [16], [18]. In children with mitochondrial respiratory disorders in the central nervous system and neuromuscular tissues, high mortality and poor prognosis are pronounced when MCM is accompanied [19], [20]. Researches using mouse models harboring genetic mutations of mitochondrial proteins has provided insights to understand the molecular basis, progression and diversity of mitochondrial respiratory disorders [21].

The cardiac muscle, which shows the highest mitochondrial abundance across tissues [3], is an excellent system to study the physiological role of a mitochondrial protein. To investigate the function of Crif1 in mouse heart, *Crif1^f/f^* mice were crossed with *Myh6-cre/Esr1* and *Ckmm-cre* transgenic mice [22], [23]. In *Myh6-cre/Esr1;Crif1^f/f^* mice, Crif1 was deleted in adult cardiomyocytes in a tamoxifen dependent manner, and these mice suffered from progressive hypertrophy and died from heart failure. Oxygen consumption and ATP production rates, COX/SDH activities and electron microscopy demonstrated that Crif1 loss affects both mitochondrial respiration and structure. In *Ckmm-cre;Crif1^f/f^* mice, Crif1 was undetectable in cardiac muscle at postnatal day 10, and the mutant mice died in two weeks postnatal, showing cardiac hypertrophy associated with mitochondrial dysfunction. We suggest these two cardiac muscle-specific Crif1 deletion mice, *Ckmm-cre;Crif1^f/f^* and *Myh6-cre/Esr1;Crif1^f/f^* mice, as animal models for early and late onset MCM, respectively.

## Materials and Methods

### Ethics Statement

This research is approved by the Seoul National University Institutional Animal Care and Use Committees (Approval number: SNU 081002-2). All animal experiments were performed according to the guidelines from the SNU IACUC and the NIH principles for the Care and Use of Laboratory Animals.

### Mice

*Myh6-cre/Esr1* and *Ckmm-cre* mice were purchased from Jackson Laboratories. *Crif1^f/f^* mice were previously generated in our laboratory [6] and backcrossed for 8 generations to C57BL/6 mice. Mice were maintained in our animal colony under institutional guidelines. *Crif1^f/f^* mice were crossed with *Myh6-cre/Esr1* to generate *Myh6-cre/Esr1; Crif1^f/f^* and *Crif1^f/f^* for the littermate control. Tamoxifen was dissolved in corn oil and administered to each mouse at 20 mg/kg by intraperitoneal injections once a day, for 4 days/round, for total 2 rounds. The 4 day interval between the rounds was to avoid the accumulation of tamoxifen inside the body from prolonged treatment. Tamoxifen injection started when mice reached 6 weeks postnatal. *Ckmm-cre;Crif1^+/f^* mice were crossed with *Crif1^f/f^* to generate *Ckmm-cre;Crif1^f/f^* and the littermate controls.

### Heart Histology, SDH and COX Activity Stains

Mouse hearts were arrested with 1 M KCl, fixed in 3.7% formalin, paraffin-embedded, and sectioned at 4 µm. The sections were subjected to hematoxylin and eosin (H&E) staining. For succinic dehydrogenase (SDH) and cytochrome c oxidase (COX) stain, excised heart were snap frozen in OCT compound (Sakura) and sectioned at 10 µm. Subsequent staining procedure was performed by standard protocols [24]. The staining intensity was quantified using ImageJ program. Microscope images were converted to grayscale and then inverted. Pixels between the threshold 95∼255 were selected to avoid the background signal, and the integrated densities were measured.

### Western Blot

Protein samples were prepared from excised hearts, and Western blot was performed by standard protocols. Primary antibodies used were: anti-Crif1 [6], and anti-actin (Sigma).

### Ecocardia

Echocardiographic measurements were performed as described previously [25]. Briefly, mice were anesthetized and echocardiographed using a Powervision 6000 (TOSHIBA) instrument with a 12-MHz microprobe (PLM-1204AT, TOSHIBA). Hearts were scanned using the M-mode guided by a short-axis view of the 2-dimensional mode.

### Mitochondrial Respiration and ATP Measurements

Cardiac muscle fibers were saponin-permeablized [26], and respiratory parameters were measured according to previously published procedures [26], [27]. Briefly, 2–3 mg of the wet tissue was transferred into Oxygraph chamber (Ocean Optics Instruments) containing mitochondrial respiration buffer to measure the oxygen concentration. Respiratory rates were expressed as nmol of O_2_/min/mg dry fiber weight. Substrates used were: 10 mmol/l pyruvate and 5 mmol/l malate; 0.02 mmol/l palmitoyl-carnitine and 2 mmol/l malate; or 5 mmol/l glutamate and 2 mmol/l malate. 1 mmol/l ADP was included to stimulate state 3 respirations. Subsequently, 8 mmol/L cytochrome c was added, and finally 1 ug/mL oligomycin were added to evaluate state 4 respirations. ATP concentrations were determined using the ATP assay kit (BioVision).

### Transmission Electron Microscopy

Cardiac tissue was fixed overnight in 2% paraformaldehyde and 2% glutaraldehyde in 0.05 M sodium cacodylate buffer (pH 7.2) at 4°C. Samples were rinsed with sodium cacodylate buffer, postfixed with 1% osmium tetroxide at 4°C for 2 hrs. After rinsing with distilled water, samples were en bloc stained with 0.5% uranyl acetate at 4°C for overnight. After the dehydration process with ethanol series, samples were transferred to propylene oxide, and embedded in Spurr’s resin. After polymerization, ultrathin sections were prepared with an ultramicrotome (MT-X, RMC, Tucson, AZ, USA). Images were captured using LIBRA 120 (Carl Zeiss, Germany). All process was performed at the EM facility of NICEM at Seoul National University.

### Cell Culture

E13.5 embryos of *Crif1^+/+^* and *Crif1^f/f^* were digested with trypsin/EDTA to isolate *Crif1^+/+^* and *Crif1^f/f^* MEFs. SV40 large T antigen was transfected into MEFs for immortalization. Cells were grown in DMEM (Hyclone) supplemented with 10% FBS (Hyclone) and antibiotic-antimycotic (Gibco) in a humidified incubator with 5% CO_2_. For Cre-retrovirus production, PT67 packaging cell line containing the retrovirus construct expressing Cre recombinase was established and maintained in standard culture medium with puromycin (3 µg/ml). When the cells become confluent, medium was changed with non-puromycin containing medium, and incubated for 24 hrs. To induce Cre mediated recombination of the floxed alleles, MEFs were incubated in Cre-retrovirus containing medium mixed with polybrene (6 µg/ml) for 24 hrs. After virus infection, cells were incubated for 36 hrs in standard medium. Virus infected MEFs were selected with puromycin (3 µg/ml) for 60 hrs until uninfected cells are fully eliminated. Chloramphenicol (Sigma) was treated at a final concentration of 100 µg/ul for 48 hrs.

### Immunofluorescence

MEFs were fixed with 3.7% formalin diluted in culture medium for 15 minutes at 37°C. After washing and blocking, cells were incubated with anti-Cox I antibody (Molecular probes), followed by incubation with anti-mouse secondary antibody. For mitochondrial stain, cells were incubated with culture medium containing 400 nM MitoTracker Red (Molecular probes) for 30 minutes at 37°C prior to fixation.

### Mitochondrial Membrane Potential

MEFs were collected by trypsinization, and resuspended in PBS containing 1% FBS and 20 mM HEPES. Fluorochrome tetramethylrhodamine ethyl ester (TMRE, Molecular probes, T669) was added to the final concentration of 100 nM for the 30 minute incubation at room temperature. For the controls of disrupted mitochondrial membrane potential, 20 µM CCCP was included in the staining solution. The fluorescence intensity was monitored in FL-2 channel and analyzed using CellQuest software (Becton Dickinson).

### Statistical Analyses

Data are presented as the mean ± SD or mean ± SE. Significance (*P*<0.05) was evaluated by a two tailed, unpaired Student’s t test.

## Results

### Mitochondrial Abnormalities in *Crif1^Δ/Δ^* MEFs

**Figure 1:**
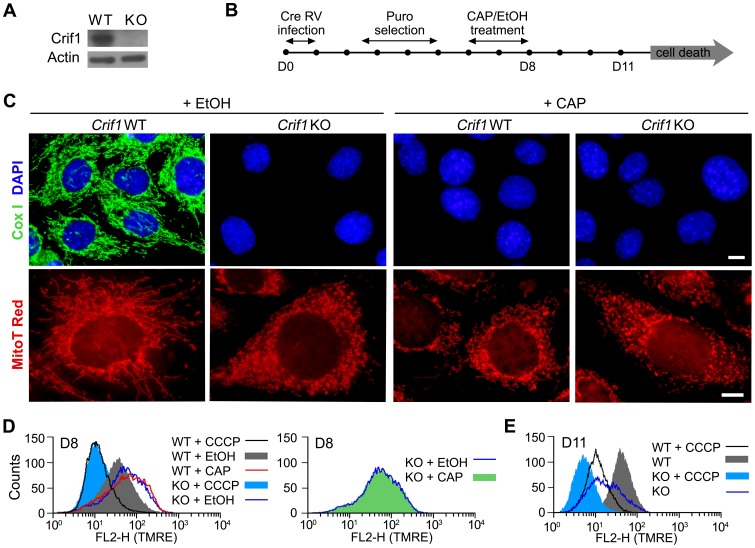
*Crif1^Δ/Δ^* and CAP treated *Crif1^+/+^* MEFs show mitochondrial abnormalities. (A) Western blot analysis of Crif1 expression in *Crif1^+/+^* and *Crif1^Δ/Δ^* MEFs. (B) Schematic presentation of experimental procedures. At day 0 (D0), MEFs were infected with retrovirus that expresses Cre recombinase. After puromycin selection, mitochondrial characteristics were analyzed after the treatment of either ethanol (EtOH) vehicle or chloramphenicol (CAP). (C) At day 8, cells were immunostained with Cox I antibody (in green) and DAPI (in blue) (upper panel), or stained with Mitotracker Red (bottom panel). Scale bars, 10 µm. (D, E) To measure mitochondrial membrane potential (MMP), MEFs were stained with tetramethylrhodamine ethyl ester (TMRE), a cationic fluorescent dye sensitive to MMP, and analyzed by flow cytometry on the FL2 channel at day 8 (D) and day 11 (E). Carbonyl cyanide *m*-chlorophenyl hydrazone (CCCP), a proton ionophore that disrupts proton gradient, was treated to gain the control peak of MMP decrease.

Crif1, which associates with the large subunit of mitochondrial ribosome, is essential for the protein synthesis of mtDNA encoded genes [10]. To demonstrate that the mitochondrial alterations in Crif1-deleted cells are caused by impaired mitochondrial translation, the mitochondria of Crif1 knockout cells were compared to that of wild type cells treated with chloramphenicol (CAP), which inhibits peptide bond formation on mitoribosomes. Immortalized *Crif1^+/+^* and *Crif1^f/f^* mouse fibroblasts were infected with Cre retrovirus and selected with puromycin to generate *Crif1^+/+^* and *Crif1^Δ/Δ^* cells, respectively. Crif1 deletion was confirmed by Western blot analysis (Fig. 1A). Changes in mitochondria were assessed in *Crif1^+/+^* and *Crif1^Δ/Δ^* cells after treating either ethanol vehicle or CAP (Fig. 1B). Immunofluorescence of Cox I, a mtDNA encoded subunit consisting respiratory chain complex IV, revealed filamentous structure of mitochondria in ethanol-treated *Crif1^+/+^*cells. The Cox I expression was completely diminished by CAP treatment, showing that CAP effectively prevents mitochondrial translation. As expected, *Crif1^Δ/Δ^* cells exhibited loss of Cox I expression regardless of CAP treatment (Fig. 1C, top panel). When the mitochondrial network was visualized by MitoTracker Red staining, *Crif1^+/+^* cells showed filamentous structure. However, the mitochondria of *Crif1^Δ/Δ^* and CAP-treated *Crif1^+/+^* cells were fragmented (Fig. 1C, bottom panel).

CAP transiently induces an increase of mitochondrial membrane potential [28]. In accordance with the previous report, *Crif1^+/+^* cells showed increased mitochondrial membrane potential by CAP treatment (Fig. 1D, red). Interestingly, the mitochondrial membrane potential in *Crif1^Δ/Δ^* cells increased to a similar extent as that in CAP-treated *Crif1^+/+^* cells (Fig. 1D, compare the red and blue lines). The increased mitochondrial membrane potential in the *Crif1^Δ/Δ^* cells was not further increased by CAP treatment (Fig. 1D, right panel) and readily depolarized by the treatment of carbonyl cyanide *m*-chlorophenyl hydrazone (CCCP), a proton ionophore (Fig. 1D). Eventually, 11 days after the gene disruption, *Crif1^Δ/Δ^* cells exhibited decrease in membrane potential, which is one of the common features of dying cells (Fig. 1E). Taken together, Crif1 loss causes mitochondrial abnormalities comparable to the effect of CAP treatment.

### Tamoxifen-inducible Deletion of Crif1 in Adult Cardiac Muscle

**Figure 2:**
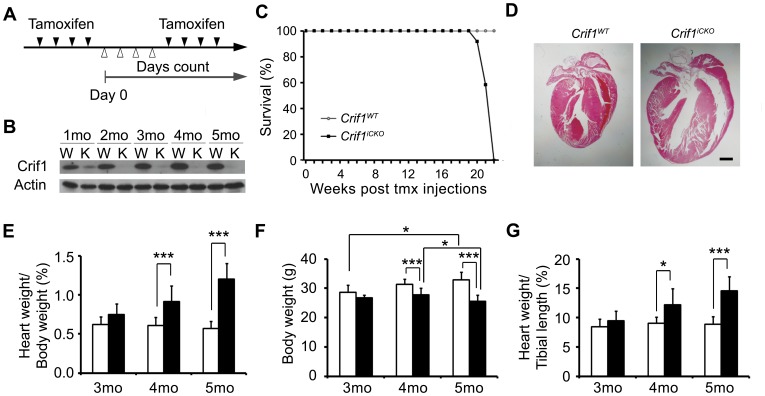
Inducible excision of Crif1 in adult cardiac myocytes causes cardiac hypertrophy. (A) Scheme of tamoxifen injections. 6 week old *Crif ^f/f^* (*Crif1^WT^*) and *Myh6-cre/Esr1;Crif^f/f^* (*Crif1^iCKO^*) mice were administrated with tamoxifen intraperitoneally at a dose of 20 mg/kg/day, for 8 days. Black arrows, days with injections. White arrows, days without injections. Day 0, start of days counted. (B) Western blot analysis of Crif1 expression in *Crif1^WT^* and *Crif1^iCKO^* hearts at 1∼5 months post tamoxifen injections (C) The survival graph of *Crif1^WT^* and *Crif1^iCKO^* mice. Survivality were checked every week after tamoxifen (txm) injections. n = 12 for both groups. (D) H&E staining of the heart sections from *Crif1^WT^* and *Crif1^iCKO^* mice at 5 months post tamoxifen injections. Scale bar, 1 mm. (E) heart weight per body weight ratio, (F) body weight and (G) heart weight per tibia length ratio of *Crif1^WT^* and *Crif1^iCKO^* mice at 3 ∼ 5 months. The tibia length of *Crif1^iCKO^* mice was not different from that of *Crif1^WT^* mice. n = 5 for each genotype at 3 months, n = 7 for *Crif1^WT^* at 4 months, n = 9 for *Crif1^iCKO^* at 4 months, n = 10 for *Crif1^WT^* at 5 months, n = 12 for *Crif1^WT^* at 5 months. Error bars show SD. ^*^ *P*<0.05, ^***^ *P*<0.005.

To investigate the physiological function of Crif1 in cardiac muscle, *Crif1^f/f^* mice were bred with *Myh6-cre/Esr1* transgenic line, which has cardiomyocyte-specific Cre recombinase activity in a tamoxifen-dependent manner [22]. *Myh6-cre/Esr1; Crif1^f/f^* and the littermate control *Crif1^f/f^* mice were administrated with tamoxifen at 6 weeks postnatal (Fig. 2A), and they are hereafter termed *Crif1^iCKO^* (Crif1 inducible cardiac KO) and *Crif1^WT^*, respectively. To verify the ablation of Crif1, we prepared the cardiac lysates from 1∼5 months post tamoxifen injections, and performed Western blot analysis using anti-Crif1 antibody. There was a significant reduction of Crif1 at one month, and it was reduced to the undetectable level at two months after the tamoxifen injections (Fig. 2B).

### Cardiac Hypertrophy in the *Crif1^iCKO^* Mice

Five months after tamoxifen treatment, *Crif1^iCKO^* mice started to die, and none of them survived more than 22 weeks (Fig. 2C). The moribund *Crif1^iCKO^* mice showed cardiac enlargement (Fig. 2D). To evaluate cardiac hypertrophy in *Crif1^WT^* and *Crif1^iCKO^* mice, the ratio of heart weight-to-body weight was measured at each month after the tamoxifen treatment. At 1∼3 months, the body weight and the heart weight-to-body weight ratios of *Crif1^iCKO^* mice appeared normal. However, cardiac hypertrophy became significant at 4 months (Fig. 2E, ^***^ *P* = 0.0016). The *Crif1^iCKO^* hearts were severely hypertrophied by a 2.1 fold increase compared to the *Crif1^WT^* hearts at 5 months.

While *Crif1^WT^* mice significantly gained weight from 3 to 5 months (^*^ *P* = 0.016), the body weight of *Crif1^iCKO^* mice did not increase; rather, an apparent decrease from 4 to 5 months was observed (^*^ *P* = 0.029) (Fig. 2F). We also observed a dramatic loss of abdominal and visceral fat in the moribund *Crif1^iCKO^* mice (data not shown). Thus, the decrease in heart weight-to-body weight ratios could be due to loss of body weight. To exclude this possibility, the heart weight-to-tibia length ratios were also calculated. As shown in Fig. 2G, cardiac hypertrophy in the *Crif1^iCKO^* mice was significant at 4 months (^*^ *P* = 0.011), and one month later, the heart weight-to-tibia length ratio was increased by 1.6-fold compared to the *Crif1^WT^* ratio. From these results, we conclude that Crif1 deletion in adult cardiac muscle causes progressive cardiac hypertrophy.

### Reduced Cardiac Function Caused by Crif1 Loss

**Figure 3:**
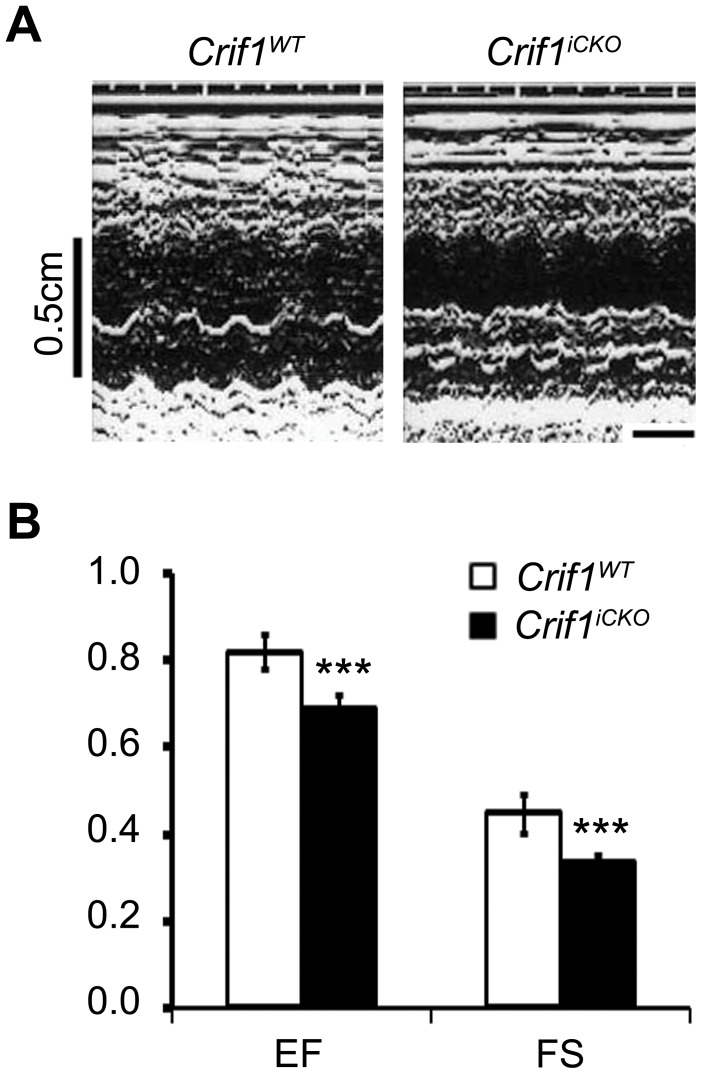
Crif1 loss impairs *in vivo* heart function. (A) Echocardiographic tracings of *Crif1^WT^* and *Crif1^iCKO^* mice at 4 months post tamoxifen injections. Scale bar = 0.1 sec. (B) Values of ejection fraction (EF) and fractional shortening (FS) obtained from echocardiography. n = 8 for *Crif1^WT^* and n = 11 for *Crif1^iCKO^* mice. Error bars show SD. ^***^ *P*<0.0001.

As *Crif1^iCKO^* mice died with cardiac hypertrophy that is often implicated in heart failure, we examined the cardiac function of *Crif1^iCKO^* mice by echocardiography at 4 months after tamoxifen treatment. The *Crif1^iCKO^* mice showed abnormal patterns of cardiac contractions when compared to the controls (Fig. 3A). In addition, the values of ejection fraction (EF) and fractional shortening (FS) were markedly decreased in the mutant mice (Fig. 3B). Hydrothorax, one of the consequences that can be caused by cardiac failure, was occasionally observed in *Crif1^iCKO^* mice at 5 months (data not shown). Thus, loss of Crif1 in cardiac muscle results in hypertrophic heart failure that leads to death.

### Impaired Mitochondrial Respiration in Crif1 Deficient Cardiac Tissues

**Figure 4:**
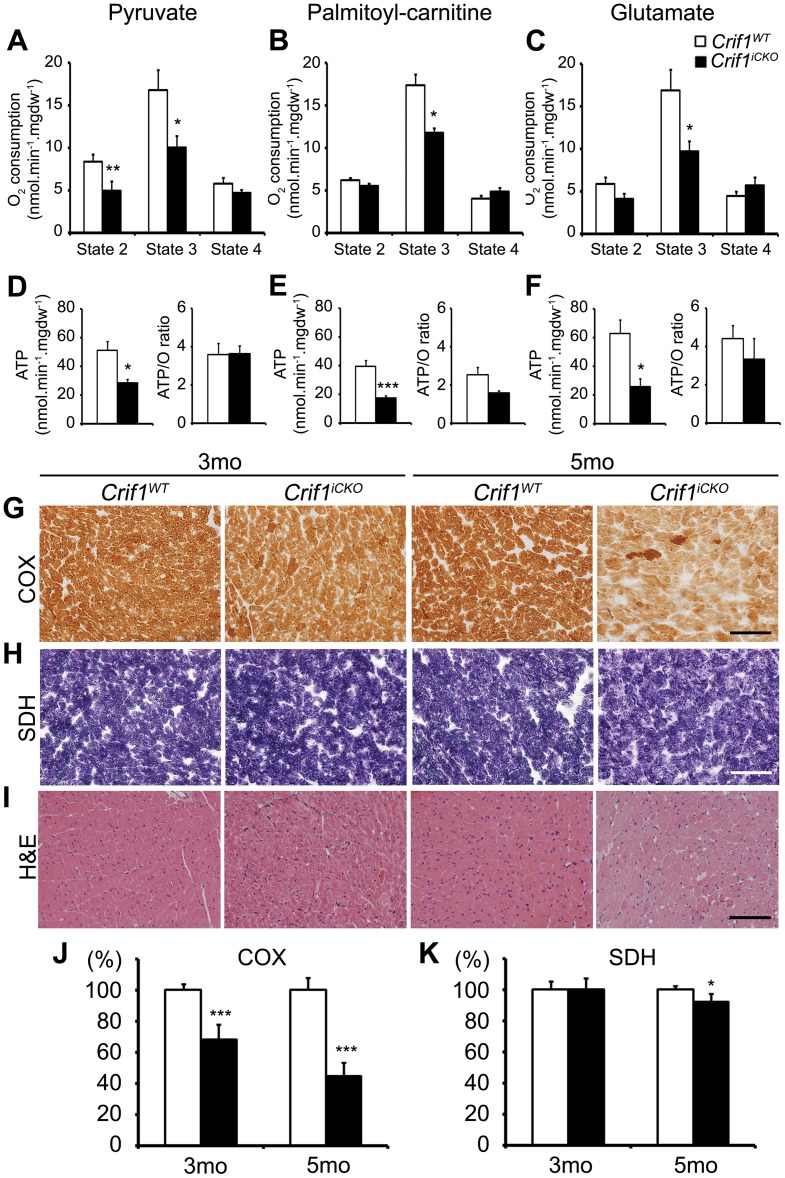
Crif1 is required for mitochondrial respiratory activities in cardiac muscle. (A∼C) Oxygen consumption rates and (D∼F) ATP synthesis rates using the saponin-permeablized cardiac fibers from *Crif1^WT^* and *Crif1^iCKO^* mice at 4.5 months post tamoxifen injections. (A, D) Pyruvate-malate respiration. n = 6 for each genotype. (B, E) PC-malate respiration. n = 6 for each genotype. (C, F) Glutamate-malate respiration. n = 5 for *Crif1^WT^* and n = 6 for *Crif1^iCKO^* mice. Error bars show SE. ^*^ *P*<0.05,^ **^ *P*<0.01,^ ***^ *P*<0.005. No significant changes were observed in ATP/O with all substrates (*P*>0.05). (G) COX, (H) SDH and (I) H&E stain on the cardiac sections of *Crif1^WT^* and *Crif1^iCKO^* mice at indicated time points. Scale bar, 100 µm. Quantification of (J) COX and (K) SDH staining intensities. Microscope images were analyzed using ImageJ software. The total intensity of the *Crif1^iCKO^* heart (black) is shown as the relative ratio to the *Crif1^WT^* stain (white). Microscope images were taken from two different sections for each heart sample. Three heart samples were collected for each genotype. Error bars show SE. ^*^ *P*<0.05,^ ***^ *P*<0.005.

Since mtDNA encodes the catalytic core subunits of respiratory chain complexes, its expression is crucial for mitochondrial respiration [29], [30], [31], [32]. To demonstrate that Crif1 is necessary for mitochondrial respiration in cardiac muscles, we measured the rate of oxygen consumption and ATP production using *Crif1^iCKO^* and *Crif1^WT^* tissues. Saponin-permeablized cardiac fibers were independently incubated with three metabolic substrates: pyruvate, palmitoyl-carnitine (PC) or glutamate. *Crif1^iCKO^* tissues incubated with pyruvate showed decreased oxygen uptake compared to *Crif1^WT^* tissues (Fig. 4A, state 2, 4.97±1.06 vs. 8.37±0.84, ^**^ *P* = 0.0086). When ADP was added in order to stimulate the maximal respiration (state 3), *Crif1^iCKO^* cardiac tissues showed significantly reduced oxygen uptake compared to *Crif1^WT^* tissues: pyruvate (Fig. 4A, 10.03±1.40 vs. 16.81±2.36, ^*^ *P = *0.017), PC (Fig. 4B, 11.77±0.51 vs. 17.35±1.31, ^*^ *P* = 0.014) or glutamate (Fig. 4C, 9.66±1.23 vs. 16.83±2.52, ^*^ *P* = 0.034). When oligomycin was added to inhibit the activity of ATP synthase (state 4), the oxygen uptake of *Crif1^iCKO^* tissues was not significantly different from that of *Crif1^WT^* tissues, indicating that uncoupled respiration is not present in *Crif1^iCKO^* tissues.

The ATP production during state 3 respiration was reduced in *Crif1^iCKO^* tissues compared to *Crif1^WT^* tissues when incubated with pyruvate (Fig. 4D, 28.46±2.47 vs. 51.00±6.16, ^*^ *P* = 0.025), PC (Fig. 4E, 17.46±1.29 vs. 39.28±4.17, ^***^ *P* = 0.0048) and glutamate (Fig. 4F, 25.59±5.79 vs. 62.68±9.69, ^*^ *P* = 0.021). For all substrates, the ratios of ATP generation to oxygen consumption (ATP/O) in *Crif1^iCKO^* tissues were insignificantly different from those of *Crif1^WT^* tissues. These data show that Crif1 is indispensible for mitochondrial respiration.

To further analyze the effect of Crif1 loss on mitochondrial respiration, the activities of respiratory chain complexes were examined by histochemical methods [24]. Serial sections of *Crif1^WT^* and *Crif1^iCKO^* hearts were stained for succinate dehydrogenase (SDH, respiratory chain complex II) and cytochrome oxidase (COX, respiratory chain complex IV) activities. The COX activity in *Crif1^iCKO^* sections decreased (68% of the *Crif1^WT^* staining intensity) at 3 months and was dramatically reduced (45% of the *Crif1^WT^* staining intensity) at 5 months (Fig. 4G and J). In contrast, the SDH activity in *Crif1^iCKO^* sections was unaffected at 3 months and showed only a subtle decrease (92% of the *Crif1^WT^* staining intensity) at 5 months (Fig. 4H and K). These results indicate that the activity of COX is progressively diminished by Crif1 loss whereas the activity of SDH, the respiratory complex exclusively composed of nuDNA encoded subunits [29], [33], is less affected in *Crif1^iCKO^* heart.

*Crif1^WT^* and *Crif1^iCKO^* heart sections were also stained with hematoxylin/eosin to examine histological abnormalities (Fig. 4I). *Crif1^iCKO^* hearts appeared normal at 3 months. However, most of the *Crif1^iCKO^* fibers were distinguished by faint eosin staining at 5 months (Fig. 4C). Tunnel staining was performed using the adjacent sections to determine necrotic fibers. However, no dying cells were observed (data not shown), indicating that the loss of mitochondrial respiration and cardiac contractility of *Crif1^iCKO^* hearts is not attributed to the death of cardiac myocytes.

### Increased Mass of Abnormal Mitochondria with Lack of Cristae in Crif1 Deficient Hearts

**Figure 5:**
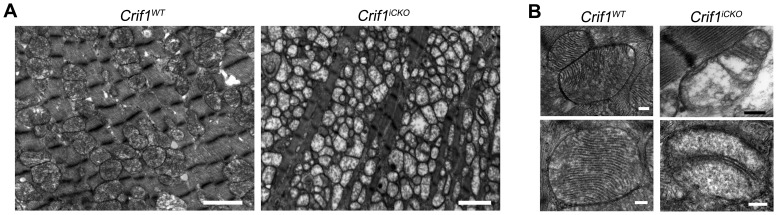
Crif1 loss increases the mass of abnormal mitochondria. (A) Low and (B) high magnification images of transmission electron microscopy. Experiments were performed using the cardiac apex from *Crif1^WT^* and *Crif1^iCKO^* mice at 5 months post tamoxifen injections. Scale bar, 2 µm (A) and 0.2 µm (B).

Aberrant mitochondrial structure in cardiomyocytes is one of the characteristic features in MCM patients [19], [20], [21]. To examine the possibility that Crif1 loss does not only affects mitochondrial respiration but also the structure, cardiac tissues were analyzed by transmission electron microscopy. Several dramatic changes in the ultrastructure were observed: decreased mass of myofibrils, increased number of mitochondria, and abnormal inner structure of mitochondria (Fig. 5A). The *Crif1^WT^* tissue exhibited mitochondria with densely packed cristae, which is a distinct characteristic of cardiac muscle. However, the structure of increased mitochondria in *Crif1^iCKO^* hearts was significantly with loss of cristae (Fig. 5B). Thus, Crif1 is required to maintain the normal mitochondrial structure.

### Cardiac Hypertrophy in *Ckmm-cre; Crif1^f/f^* Mice

**Figure 6:**
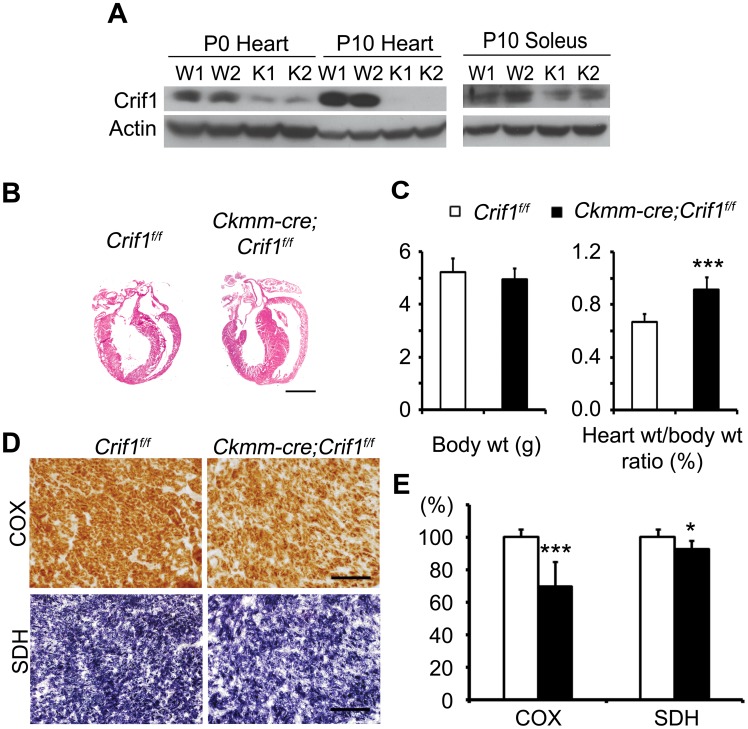
Crif1 deletion in the cardiac muscle using the *Ckmm-cre* transgene leads to cardiac hypertrophy. (A) Western blot analysis of Crif1 expression in isolated hearts and soleus muscles from *Crif1^f/f^* and *Ckmm-cre;Crif1^f/f^* mice. Protein lysates were prepared from P0 or P10 mouse tissues. (B) H&E staining of heart sections from *Crif1^f/f^* (W1 and W2) and *Ckmm-cre;Crif1^f/f^* mice (K1 and K2) at P10. Scale bar, 1 mm. (C) Heart weight per body weight ratios of P10 mice. n = 7 for each genotype. Error bars show SD. ^***^ *P* = 0.00001. (D) COX and SDH stain on snap-frozen P10 heart sections. Scale bars, 100 µm. (E) Quantification of COX and SDH staining intensities using ImageJ. The total intensity of the *Ckmm-cre;Crif1^f/f^* heart (black) is shown as the relative ratio to the *Crif1^f/f^* stain (white). Microscope images were taken from two different sections for each heart sample. Three heart samples were collected for each genotype. Error bars show SE. ^*^ *P*<0.05,^ ***^ *P*<0.005.

Crif1 was not only ablated in the adulthood, but was also ablated in neonatal cardiac muscle by using *Ckmm-cre* transgenic line, the promoter of which is active in the cardiac and skeletal muscle since embryonic day 13 [23]. Western blot analysis showed that Crif1 was reduced at P0 and undetectable in hearts at P10. Crif1 was residual in soleus muscles, which is a type of highly oxidative skeletal muscle (Fig. 6A). The mice were born in Mendelian ratios, but *Ckmm-cre; Crif1^f/f^* mice all died before postnatal day 14 (P14). We assessed COX and NADH activity of P10 *Ckmm-cre; Crif1^f/f^* soleus muscle by histochemical staining, but no decrease in these activities was observed compared to the wild type mice (data not shown). In contrast, the *Ckmm-cre;Crif1^f/f^* mouse hearts displayed morphological changes: thickened interventricular septum wall and enlarged right ventricle (Fig. 6B). The heart weight per body weight ratio of *Ckmm-cre;Crif1^f/f^* mice was higher than that of the control mice by 37.4%, showing significant hypertrophy (Fig. 6C). To test the mitochondrial respiratory activities in the young cardiac tissues, COX and SDH staining were performed (Fig. 6D and E). The decrease in COX activity in *Ckmm-cre;Crif1^f/f^* cardiac muscles (69% of the wild type staining intensity) revealed mitochondrial dysfunction in those tissues. In conclusion, the Crif1 conditional knockout mice generated by using the two different *cre* transgenic lines, *Ckmm-cre* and *Myh6-cre/Esr1*, develop cardiomyopathy due to impaired mitochondrial respiration.

## Discussion

Crif1 has been shown to regulate the function of diverse nuclear proteins and transcriptional factors [5], [6], [7], [8], [9]. However, a recent study demonstrated that Crif1 is a novel interacting factor of the mitochondrial ribosome [10]. According to this report, Crif1 is essential for mitochondrial translation and membrane integration of the respiratory chain subunits. The protein levels of mtDNA encoded proteins were diminished in Crif1 null MEFs, thus decreasing the amount of respiratory chain complexes. It seems that the mitochondrial function of Crif1 is independent of its nuclear function, as the previous report demonstrates the mitochondrial translation defect of Crif1 null MEFs is only rescued by over-expressing the C-terminal tagged construct, and not by the nuclear-localized construct, of which the signal peptide is masked by N-terminal tagging [10]. In this study, we further investigated the mitochondrial changes in Crif1 null MEFs. Not only reduced expression of Cox I, but also fragmented mitochondrial network and the membrane potential change were observed in Crif1 null MEFs. Intriguingly, these alterations were identically induced in wild type cells by CAP treatment. Furthermore, we observed a dramatic loss of cristae in the Crif1 deleted cardiac tissues. It is well known that CAP treatment reduces the number of cristae in yeast, protozoa, and mammalian cells [34], [35], [36], [37], [38]. Our results strongly suggest that the various mitochondrial alterations in Crif1 cells are commonly resulted from impaired mitochondrial translation.

While approximately half of the mitochondrial proteome shows tissue-specific expression [3], Crif1 is one of the mitochondrial proteins that are ubiquitously detected throughout the mouse organs [2]. Various conditional knockout mice of Crif1 have been generated to investigate the physiological function of Crif1; however, up to date, there is only one report demonstrating its mitochondrial function *in vivo* [5], [6], [10]. To further elucidate the mitochondrial function of Crif1, we ablated the gene in the cardiac muscle, where the mitochondrial function is critical for the organ performance. *Myh6-cre/Esr1* transgenic line was crossed with Crif1 conditional knockout mice to generate *Crif1^iCKO^* mice. The *Crif1^iCKO^* mice developed severe cardiac hypertrophy, and died within six months after the tamoxifen injections. Decreased *in vivo* heart function was demonstrated by echocardiography. The heart weight-to-tibia length ratio shows a mild cardiac enlargement (1.3-fold increase) in *Crif1^iCKO^* mice at 4 months when the echocardiography was performed. Our result suggests that the cardiac function decreases from the early point of hypertrophy in *Crif1^iCKO^* mice. The rate of substrate-driven respiration using permeablized cardiac tissues revealed no sign of uncoupled respiration in *Crif1^iCKO^* heart, but the rate of oxygen uptake and ATP generation were significantly reduced. *Crif1^iCKO^* hearts show a mild decrease in COX activity at 3 months post tamoxifen injections, and it takes two more months to show a dramatic change. These results indicate that the initial mitochondrial defect in Crif1 deleted cells is relatively subtle but gradually aggravates to more severe disruption. In addition, the electron microscopy revealed a dramatic change in mitochondrial morphology in *Crif1^iCKO^* cardiac tissues.

Our study shows that Crif1 loss brings out a series of mitochondrial damages in membrane potential, respiratory activities and structure. Thus, Crif1 is considered as an essential factor for mitochondrial homeostasis and integrity by playing its primary function in mitochondrial translation. Since each cell type has its own mitochondrial/cytosolic proteome, the progression of mitochondrial dysfunction and secondary alterations of cellular metabolism will differ depending on the cell type of Crif1 deletion. While MEFs are sensitive to Crif1 loss, so that the cell death is triggered in days, adult cardiomyocytes that are specialized in energy production seems to resist against cell death. We speculate that Crif1 loss accumulates mitochondrial alterations until the cells eventually die or fail to perform its cellular function.

We also ablated Crif1 in neonatal cardiac muscle by using *Ckmm-cre* transgenic line. In accordance with the phenotypes of *Crif1^iCKO^* mice, *Ckmm-cre; Crif1^f/f^* mice exhibited cardiac hypertrophy associated with mitochondrial dysfunction. The adult cardiac deletion of Crif1 followed a gradual course of hypertrophy and longer survival, while *Ckmm-cre;Crif1^f/f^* mice showed less severe hypertrophy but early death. We speculate that the heart of *Ckmm-cre; Crif1^f/f^* mice showed a rapid functional failure in contraction (thus causing early death) before the mitochondria developed prominent pathological defects. Whereas mitochondrial dysfunction is progressively aggravated in *Crif1^iCKO^* cardiomyocytes, the cardiac function of the young heart seems to be much more vulnerable to early mitochondrial defects caused by Crif1 loss. Here we suggest one possibility how the adult heart is more resistant to Crif1 loss. *Crif1^iCKO^* cardiomyocytes might activate glycolytic pathways to compensate for the energy shortage as previously shown in Crif1 deleted MEFs [10]. The energy metabolism of fetal and immediate newborn hearts depends on glycolysis and glucose oxidization, but within 24 hours after birth, the heart rapidly switches to fatty acid oxidization for ATP production [39], [40]. Although the precise mechanism is not yet known, redirection toward fetal metabolism is well-documented as a hallmark of failing hearts, and strongly suggested as an adaptive response to increase survival [39], [40]. Thus, *Crif1^iCKO^* heart also may return to fetal metabolism. However, we speculate that *Ckmm-cre; Crif1^f/f^* hearts fail to reinforce the fetal metabolism when the massive change in gene expression profile is triggered by birth. Crif1 loss is surely a stress for cardiomyocytes, but the ultimate extrinsic signal for the fetal heart is birth [41]. Not only the metabolism, but also other differences between young and adult cardiomyocytes, such as myosin isoform composition [39], may contribute to the phenotypic differences in young and adult cardiac deletion models of Crif1.

The late onset mitochondrial respiratory disorders tend to follow a chronic course, and the early onset respiratory disorders show more severe clinical presentations [12], [14]. How the “age-dependent” molecular context affects the disease progression has not yet been investigated. We suggest the two different cardiac specific Crif1 deletion mouse models, *Ckmm-cre; Crif1^f/f^* and *Crif1^iCKO^* mice, as early (young) onset and late (adult) onset MCM models, respectively. Further comparative analysis using *Ckmm-cre; Crif1^f/f^* and *Crif1^iCKO^* mice may provide insights to understand the progression of mitochondrial cardiomyopathy.
